# Metabolic enhancers supporting 1-carbon cycle affect sperm functionality: an *in vitro* comparative study

**DOI:** 10.1038/s41598-018-30066-9

**Published:** 2018-08-06

**Authors:** A. Gallo, Y. Menezo, B. Dale, G. Coppola, M. Dattilo, E. Tosti, R. Boni

**Affiliations:** 1 Department of Biology and Evolution of Marine Organisms, Stazione Zoologica Anton Dohrn, 80121 Napoli, Italy; 2 London Fertility Associates, 104 Harley Street, London, United Kingdom; 3 Laboratoire Clement, Paris, France; 4 Centre for Assisted Fertilization, Naples, Italy; 5 Parthenogen, Lugano, 6900 Switzerland; 6 Department of Sciences, University of Basilicata, 85100 Potenza, Italy

## Abstract

The sperm plasma membrane is a sensitive target to oxidative stress. The most representative reactive oxygen species (ROS) scavengers in the genital tract, hypotaurine and glutathione, require, for their synthesis, cysteine whose availability is associated with the 1-carbon cycle (1-CC). Human, bovine and ascidian spermatozoa were incubated with compounds supporting the 1-CC (Vitamin B6, Methylcobalamin, 5 Methyl Tetrahydrofolate, Zinc Bisglycinate and N-acetyl-cysteine) (TRT) and compared to the effects induced solely by N-acetyl-cysteine (NAC). In control groups (CNTRL), spermatozoa were incubated with medium alone. After 90 and 180 minutes of incubation, the mitochondrial membrane potential (ΔΨM) in TRT and NAC was significantly (P < 0.01) higher than in CNTRL. At H_2_DCFDA evaluation, ROS production differed between species whereas, at 2-OH Ethidium, it significantly decreased in bovine TRT group. Intracellular pH (pH_i_) did not significantly vary in relation to treatment. In ascidian spermatozoa, the NAC supplementation decreased external pH, which in turn brought to a pH_i_ lowering. Buffering seawater with NaHCO_3_ reversed the beneficial effects of N-acetyl-cysteine supplementation. In conclusion, both fully supporting the 1-CC and treatment with N-acetyl-cysteine alone improved kinetics, ΔΨM and ROS production in mammalian sperm demonstrating for the first time the direct *in vitro* effects of these compounds on sperm functionality.

## Introduction

The spermatozoon is a highly differentiated cell with a compacted nucleus, scarce cytoplasm, mitochondria and usually a flagellum. The mitochondria generate energy mainly for motility, via oxidative phosphorylation, and this depends on external sources of carbohydrate^1^ and fatty acids with a consequent production of large amounts of NADH and FADH_2_. In the mitochondria, electrons move from NADH to O_2_ via enzymatic complexes, such as NADH-Q oxidoreductase, Q-cytochrome C oxido-reductase, the cytochrome C oxidase, and the complex V: ATP synthase. However, approximately 1–4% of oxygen escapes and forms reactive oxygen species (ROS)^2^. The oxidases generating free radicals affect mitochondrial structure and activity leading to cell aging^3^. ATP production and leakage of ROS by the electron transport chain are dependent on the mitochondrial membrane potential (ΔΨM)^4,5^, by measuring which the mitochondrial energy metabolism can be monitored. The ΔΨM is, however, affected by other metabolic processes such as calcium uptake and anti-oxidant defenses^6,7^.

The high mitochondrial activity of spermatozoa together with the small amount of cytoplasm and a high concentration of polyunsaturated fatty acids in plasma membrane makes these cells extremely sensitive to oxidative damage, which may affect DNA, proteins and membrane integrity. In the latter case, motility^8^, adhesion and fusion during fertilization process and the acrosome reaction may be strongly impaired^9,10^. Based on these observations, the idea that antioxidant molecules might be helpful for sperm protection and storage is of current interest. Many studies have been carried out using a variety of antioxidants for improving sperm quality^11^. In previous studies, nucleophilic thiols were used to counteract the effects of ROS^12,13^ on sperm activity and prevention of DNA adducts; however, their roles on mitochondrial functionality were not evaluated.

Glutathione (GSH) is the universal cell ROS scavenger in cells; whereas, hypotaurine is the most representative ROS scavenger in the genital tract^14^. Both these molecules require cysteine for their synthesis^15^. Cysteine availability is associated with the one carbon cycle (1-CC), as homocysteine, resulting from methionine de-methylation that can be regenerated to cystathionine by the trans-sulfuration pathway leading to the formation of cysteine (Supplemental Fig. S1). Methionine is a mandatory metabolite in the methylation processes. Within the 1-CC, it is transformed by the methionine adenosyl transferase to form the universal methyl donor S-adenosylmethionine (SAM). SAM is the cofactor of the methyl transferases releasing methyl tags on proteins (histones), nucleic acids and lipids to form phospholipids^16^. The byproducts of these reactions, i.e., S-adenosylhomocysteine (SAH) and, then, homocysteine, must be recycled to methionine since homocysteine is an inhibitor of methylation and a cellular poison at high concentrations^17^. Similarly, vitamin B12 and B6 are compulsory cofactors in this methionine regeneration activity^18^. The betaine-homocysteine S-methyltransferase (BHMT) pathway has a weaker regeneration activity^19^. In addition, SAM is also necessary for the synthesis of Coenzyme Q10, a lipid-soluble, powerful antioxidant representing an essential cofactor in mitochondrial oxidative phosphorylation^20^.

In addition to energy availability, intracellular pH (pH_i_) can strongly affect sperm functionality. This is mainly regulated via defined mechanisms: the Na^+^/H^+^ antiport (NHE), Na^+^-HCO^3−^-(NaCO^3−^) symport, and Cl^−^/HCO3^−^ exchange (AE2)^21^. Ionic transport is strongly linked to the hydrolysis of ATP. Hence, pH_i_ maintenance is an energy consuming activity requiring a highly efficient energy production also needed for the transport of nutrients used as energy sources. This intra-membrane transport can be seriously impaired by lipid peroxidation produced by ROS.

The enhancement of sperm energy metabolism is, therefore, a topic of enormous interest linked to the possibility of improving sperm functionality and providing more in-depth tools and knowledge for the management of sperm conservation programs. In this line, this study aimed to evaluate the efficacy of a treatment of fully supporting the 1-CC on sperm functionality. In particular, the effects of five compounds involved in the 1-CC, i.e., Vitamins B6 and methyl B12, 5 methyl folate, zinc and N-acetyl cysteine (NAC) on different sperm parameters, such as mitochondrial activity, lipid peroxidation, ROS production and intracytoplasmic pH, were evaluated in human, bovine and the ascidian *C. robusta*. This treatment was compared to the impact of NAC alone, since in preliminary trials (Supplemental Fig. S2) this compound showed a predominant activity compared to that of the other individually evaluated enhancers.

## Results

### Sperm kinetics

**Figure 1:**
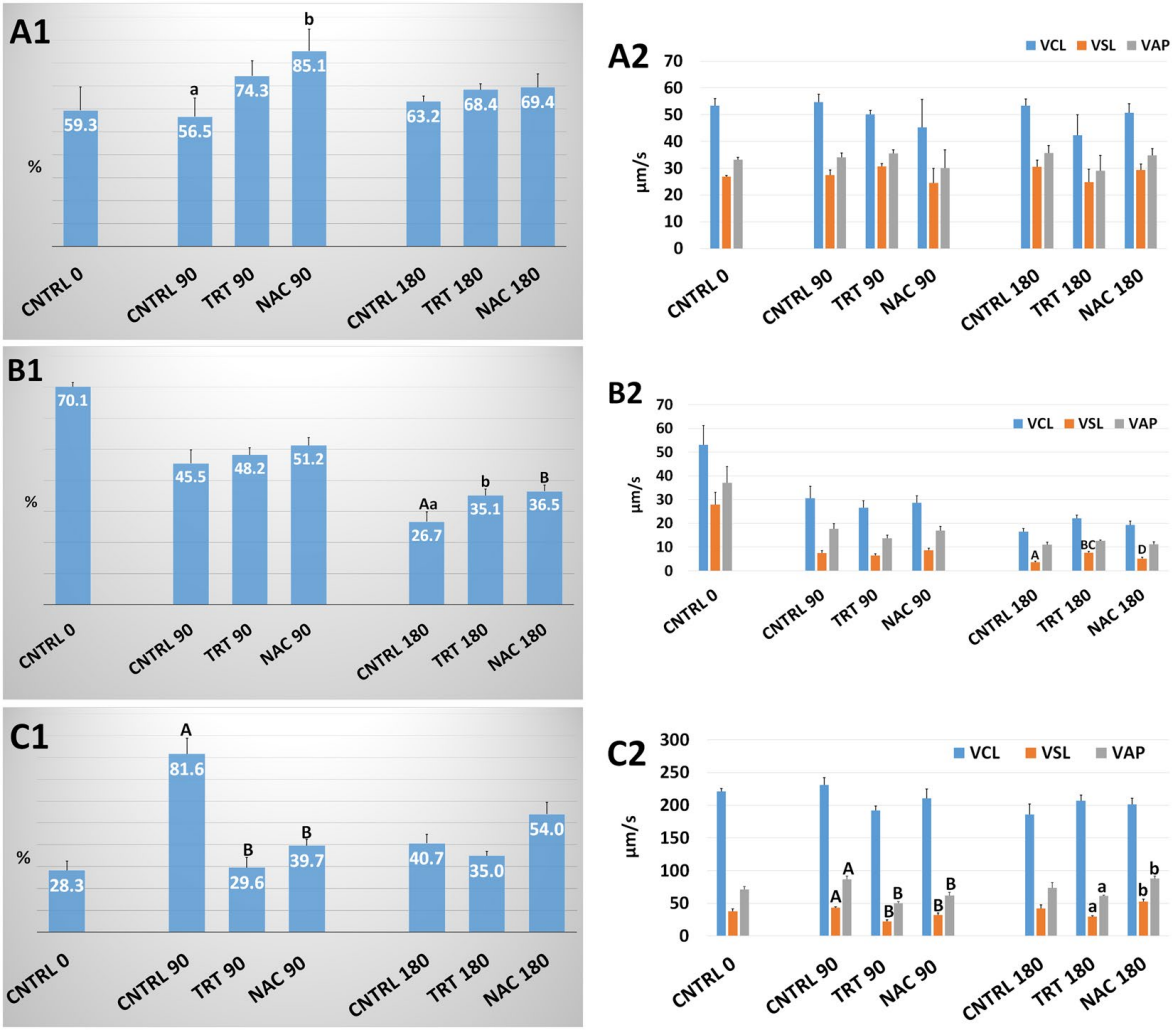
Human, bovine and ascidian sperm kinetics following exposure to metabolic enhancers. Total sperm motility and kinetics (VCL, VSL and VAP) in human (**A1**,**A2**), bovine (**B1**,**B2**) and ascidian (**C1**,**C2**) sperm divided in three groups treated with: (1) five metabolic enhancers (vitamins B6 and B12, 5 methyl THF, zinc and N-acetyl cysteine) (TRT group); (2) n-acetyl-cysteine (NAC group); and (3) medium alone (CNTRL group). Mean (± SE) values in CNTRL, TRT and NAC groups after 0, 90 and 180 min incubation. Statistically significant differences between groups for each evaluated time point are expressed as different capital (A *vs*. B and C *vs*. D; P < 0.01) and small (a *vs*. b; P < 0.05) letters.

In the human, we observed (Fig. 1A1) a significant improvement in sperm motility at 90 min incubation in NAC group in comparison with CNTRL (85.1 ± 9.5% *vs*. 56.5 ± 8.2%; P < 0.05). This beneficial effect, however, decreased at 180 min. Kinetic parameters did not show significant differences between groups neither at 90 min nor at 180 min incubation (Fig. 1A2).

In bovine, a progressive time-dependent decrease in sperm motility was observed in all the groups evaluated (Fig. 1B1). Both the TRT and NAC treatments significantly improved total motility after 180 min incubation with respect to CNTRL (35.1 ± 2.1% *vs.* 26.7 ± 3.2; P < 0.05 and 36.5 ± 2.0% *vs*. 26.7 ± 3.2%;P < 0.01). However, the beneficial effects observed in TRT and NAC groups were not sufficient to effectively counteract the natural decline of motility which was inferior to CNTRL at time 0 even after 90 min incubation (48.2 ± 2.3% and 51.2 ± 2.5% *vs*. 70.1 ± 1.4%; P < 0.01). Regarding kinetic indicators, only VSL values were significantly (P < 0.01) higher in TRT than in CNTRL whereas NAC was lower (P < 0.01) than TRT but it did not differ from CNTRL (Fig. 1B2).

In the ascidian, the overall sperm motility significantly decreased in TRT and NAC *vs*. CNTRL after 90 min incubation (29.6 ± 4.6% and 39.7 ± 3.0% *vs*. 81.6 ± 7.3%; P < 0.01) (Fig. 1C1). However, after 180 min incubation both treatments did not differ with respect to the CNTRL. Also the sperm kinetic parameters (Fig. 1C2), as VSL and VAP, at 90 min were lower in TRT and NAC than CNTRL groups (VSL = 22 ± 2 and 32 ± 3 *vs.* 43 ± 2 µm/s; P < 0.01) (VAP = 50 ± 3 and 62 ± 5 vs. 87 ± 5 µm/s; P < 0.01). After 180 min incubation, however, VSL and VAP values of TRT and NAC did not differ from CNTRL but NAC showed higher values than TRT (VSL = 52 ± 4 *vs.* 30 ± 1 µm/s; P < 0.05) (VAP = 88 ± 3 *vs*. 61 ± 1 µm/s; P < 0.05).

### Mitochondrial Membrane Potential (ΔΨM)

**Figure 2:**
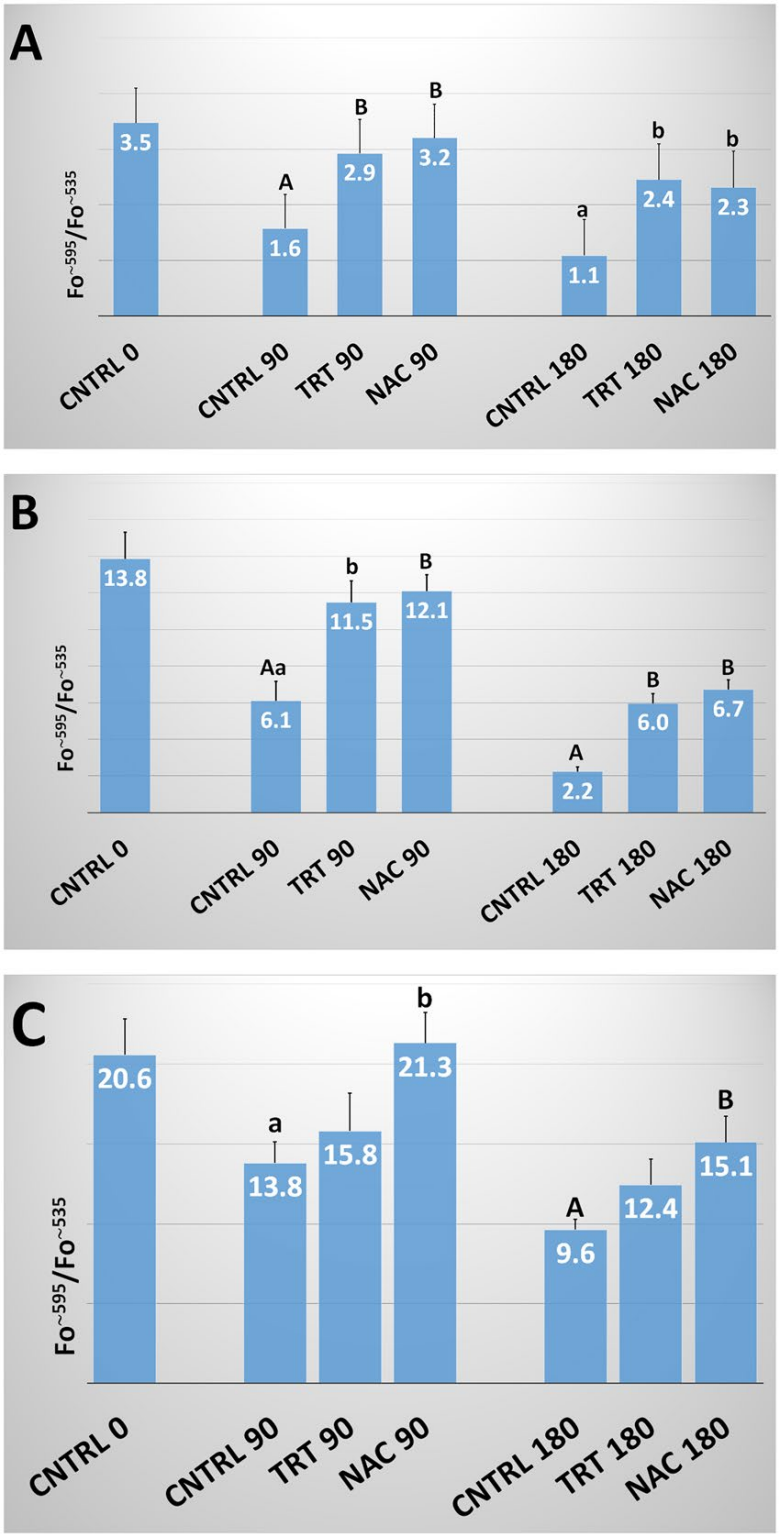
ΔΨM in human, bovine and ascidian spermatozoa exposed to metabolic enhancers. Mitochondrial membrane potential (ΔΨM) in human (**A**), bovine (**B**) and ascidian (**C**) sperm divided in three groups treated with: (1) five metabolic enhancers (vitamins B6 and B12, 5 methyl THF, zinc and N-acetyl cysteine) (TRT group); (2) n-acetyl-cysteine (NAC group); and (3) medium alone (CNTRL group). Mean (±SE) values of ΔΨM in spermatozoa loaded with JC-1, arranged in TRT, NAC and CNTRL groups and then incubated for 0, 90 and 180 min. The fluorescence emission peak (Fo) was measured at ~595 and ~535 nm and expressed as Fo^~595^/Fo^~535^. Statistically significant differences between groups for each evaluated time point are expressed as different capital (A *vs*. B; P < 0.01) and small (a *vs*. b; P < 0.05) letters.

The incubation period significantly affected ΔΨM causing a progressive reduction in all three species. Moreover, the ΔΨM significantly increased in either TRT or NAC groups in all the species considered (Fig. 2). In human spermatozoa, at 90 min incubation, both treatments induced ΔΨM values significantly higher than those of CNTRL (2.9 ± 0.5 and 3.2 ± 0.4 *vs*. 1.6 ± 0.3; P < 0.01) and similar to those recorded in CNTRL at time 0 (3.5 ± 0.6). Even at 180 min incubation, the ΔΨM values of TRT and NAC groups were higher than CNTRL (2.4 ± 0.5 and 2.3 ± 0.4 *vs*. 1.1 ± 0.2; P < 0.05) and did not differ between themselves. Although lower than at 90 min, ΔΨM of TRT and NAC groups did not significantly differ with CNTRL at time 0.

In bovine sperm, both treatments caused a ΔΨM increase as observed in human sperm. Indeed, at 90 min incubation, the two treatments showed ΔΨM levels significantly higher than CNTRL (11.5 ± 1.2 and 12.1 ± 0.9 *vs*. 6.1 ± 1.1; P < 0.05 and P < 0.01, respectively) and similar then CNTRL at time 0 (13.8 ± 1.5). At 180 min, the two treatments were higher than CNTRL (6.0 ± 0.5 and 6.7 ± 0.5 *vs*. 2.2 ± 0.3; P < 0.01) but significantly (P < 0.01) lower than CNTRL at time 0.

In ascidian sperm, after 90 min incubation, NAC group showed significantly higher levels of ΔΨM than CNTRL (21.3 ± 1.9 *vs*. 13.8 ± 1.3; P < 0.05). At 180 min, NAC maintains a significant higher value than CNTRL (15.1 ± 1.7 *vs*. 9.6 ± 0.7; P < 0.01). At this time, only NAC maintained levels of ΔΨM that were not significantly different from the CNTRL at time 0 (15.1 ± 1.7 *vs*. 20.6 ± 2.2).

### Lipid peroxidation

**Figure 3:**
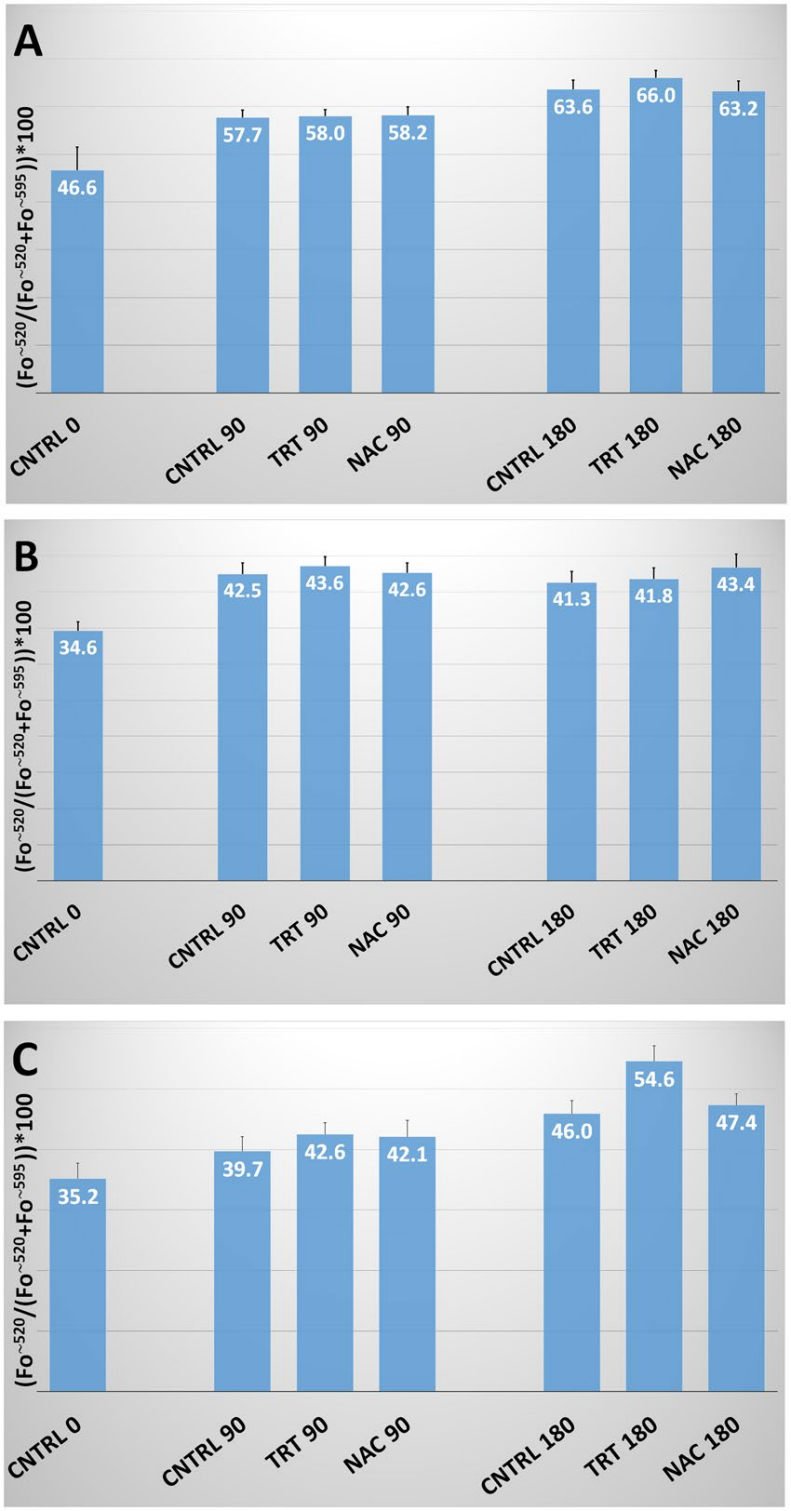
Lipid Peroxidation in human, bovine and ascidian spermatozoa exposed to metabolic enhancers. Lipid peroxidation in human (**A**), bovine (**B**) and ascidian (**C**) sperm divided in three groups treated with: (1) five metabolic enhancers (vitamins B6 and B12, 5 methyl THF, zinc and N-acetyl cysteine) (TRT group); (2) n-acetyl-cysteine (NAC group); and (3) medium alone (CNTRL group). Mean (± SE) values of lipid peroxidation in spermatozoa loaded with C11-BODIPY^581/591^, arranged in TRT, NAC and CNTRL groups and then incubated for 0, 90 and 180 min. Lipid peroxidation was calculated by relating the emission peak (Fo) at ~520 nm to the sum of the fluorescence emission peaks at ~520 and ~590 nm, i.e., ((Fo^~520^/ (Fo^~520^ + Fo^~590^)) * 100.

Lipid peroxidation, as evaluated by C11- BODIPY^581/591^ and spectrofluorometric analysis, showed a significant (P < 0.01) time-dependent increase in all the species studied. However, no significant effect was observed in the treated groups (Fig. 3).

### ROS production

**Figure 4:**
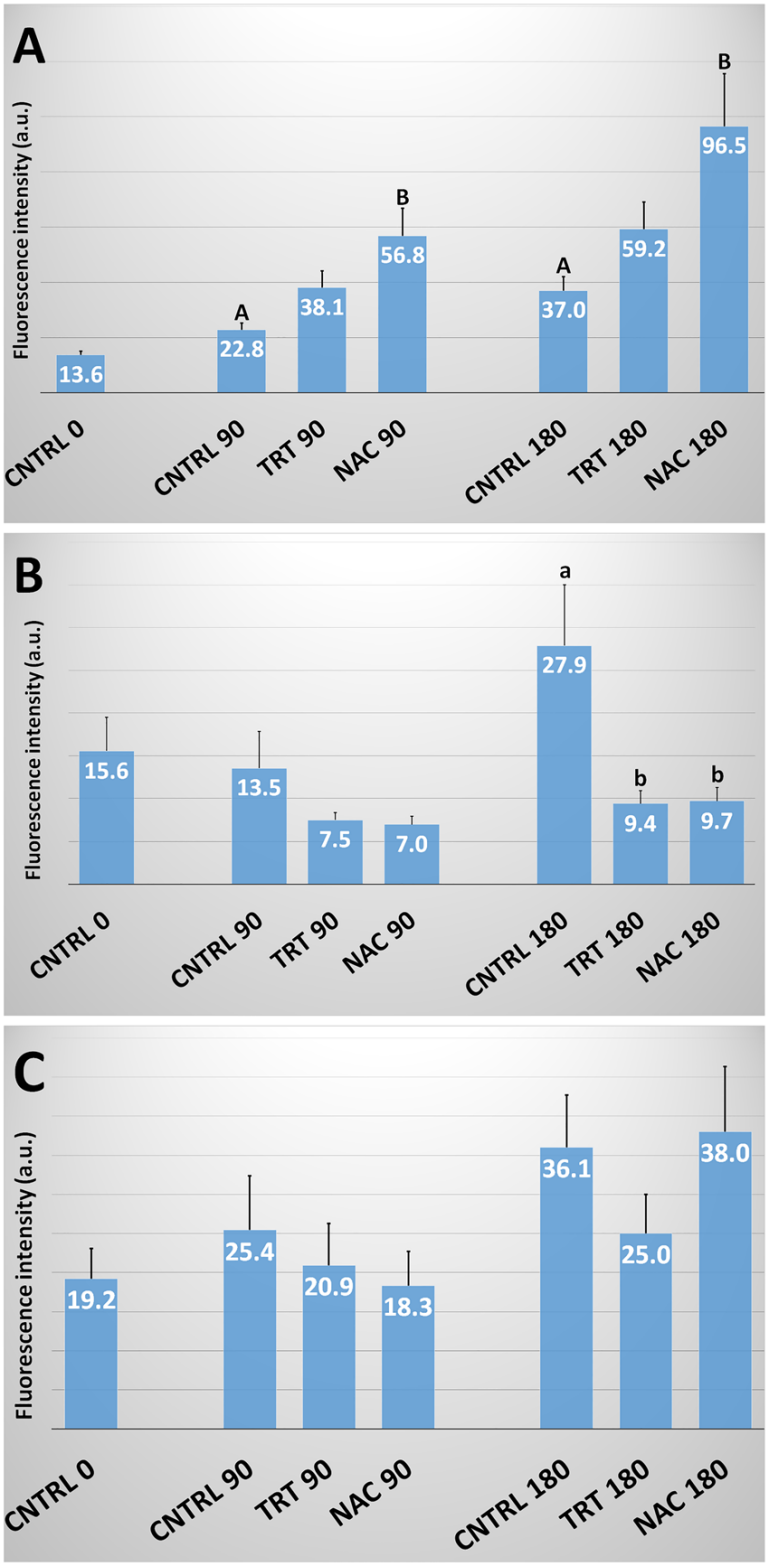
H_2_DCFDA-detected ROS in human, bovine and ascidian spermatozoa exposed to metabolic enhancers. Reactive Oxygen Species (ROS) detected by H_2_DCFDA in human (**A**), bovine (**B**) and ascidian (**C**) sperm divided in three groups treated with: (1) five metabolic enhancers (vitamins B6 and B12, 5 methyl THF, zinc and N-acetyl cysteine) (TRT group); (2) n-acetyl-cysteine (NAC group); and (3) medium alone (CNTRL group). Mean (±SE) values of ROS were evaluated in H_2_DCFDA-loaded spermatozoa, arranged in TRT, NAC and CNTRL groups and then incubated for 0, 90 and 180 min. ROS production was calculated by evaluating fluorescence intensity (Fo) at the emission peak (~520 nm). Statistically significant differences between groups for each evaluated time point are expressed as different capital (A *vs*. B; P < 0.01) and small (a *vs*. b; P < 0.05) letters.

The production of ROS, detected by H_2_DCFDA, increased with incubation time in the CNTRL of all the considered species (Fig. 4). Instead, the effects of the treatments varied according to the species. In particular, in human, ROS production significantly (P < 0.01) increased in NAC at both 90 and 180 min while TRT did not differ from CNTRL. In bovine spermatozoa, both TRT and NAC showed ROS levels significantly (P < 0.05) lower than in the CNTRL group at 180 min incubation.

Ascidian sperm did not show significant differences between groups at either 90 or 180 min incubation.

**Figure 5:**
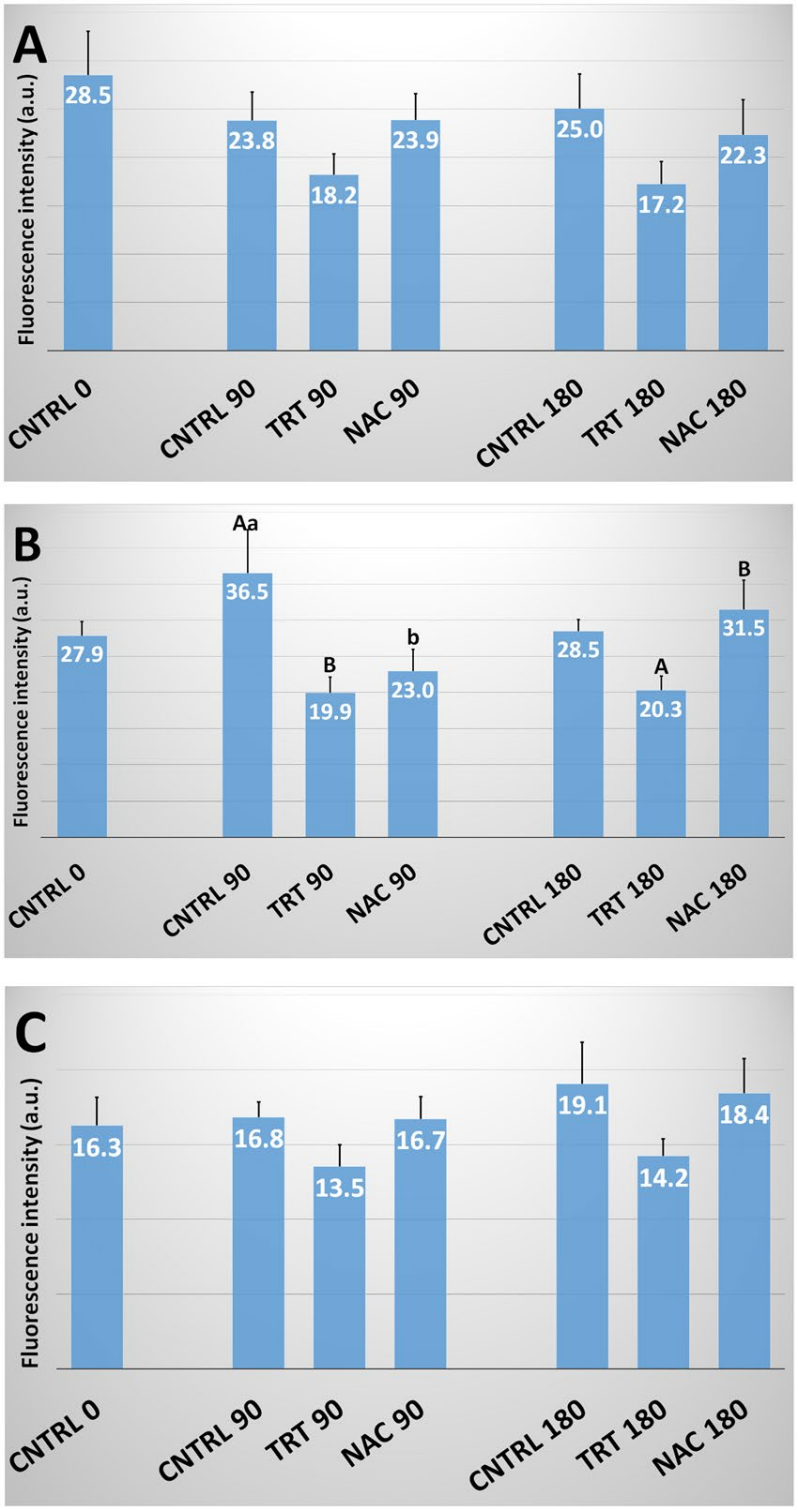
DHE-detected ROS in human, bovine and ascidian spermatozoa exposed to metabolic enhancers. Reactive Oxygen Species (ROS) detected by dihydroethidium (DHE) in human (**A**), bovine (**B**) and ascidian (**C**) sperm divided in three groups treated with: (1) five metabolic enhancers (vitamins B6 and B12, 5 methyl THF, zinc and N-acetyl cysteine) (TRT group); (2) n-acetyl-cysteine (NAC group); and (3) medium alone (CNTRL group). Mean (±SE) values of ROS production in spermatozoa loaded with DHE, arranged in TRT, NAC and CNTRL groups and then incubated for 0, 90 and 180 min. ROS production was calculated by fluorescence intensity (Fo) at the emission peak (~590 nm). Statistically significant differences between groups for each evaluated time point are expressed as different capital (A *vs*. B; P < 0.01) and small (a *vs*. b; P < 0.05) letters.

The production of ROS, detected by DHE (O_2_^−^-sensitive probe), varied among time points (Fig. 5). In human sperm, analyzing data without considering the effect of the incubation time, 2-OH E levels were significantly (P < 0.01) lower in TRT than in CNTRL and NAC groups. In bovine sperm, after 90 min incubation, the 2-OH E levels of TRT and NAC groups were significantly lower than in CNTRL group. After 180 min incubation, TRT group showed lower (P < 0.01) 2-OH E levels than NAC but it did not significant differ from CNTRL.

In ascidian sperm, the pattern of 2-OH E levels resembled those already described in human sperm; however, due to a higher variability among experiments, the comparison between groups did not show significant differences.

### Intracellular pH (pH_i_)

**Figure 6:**
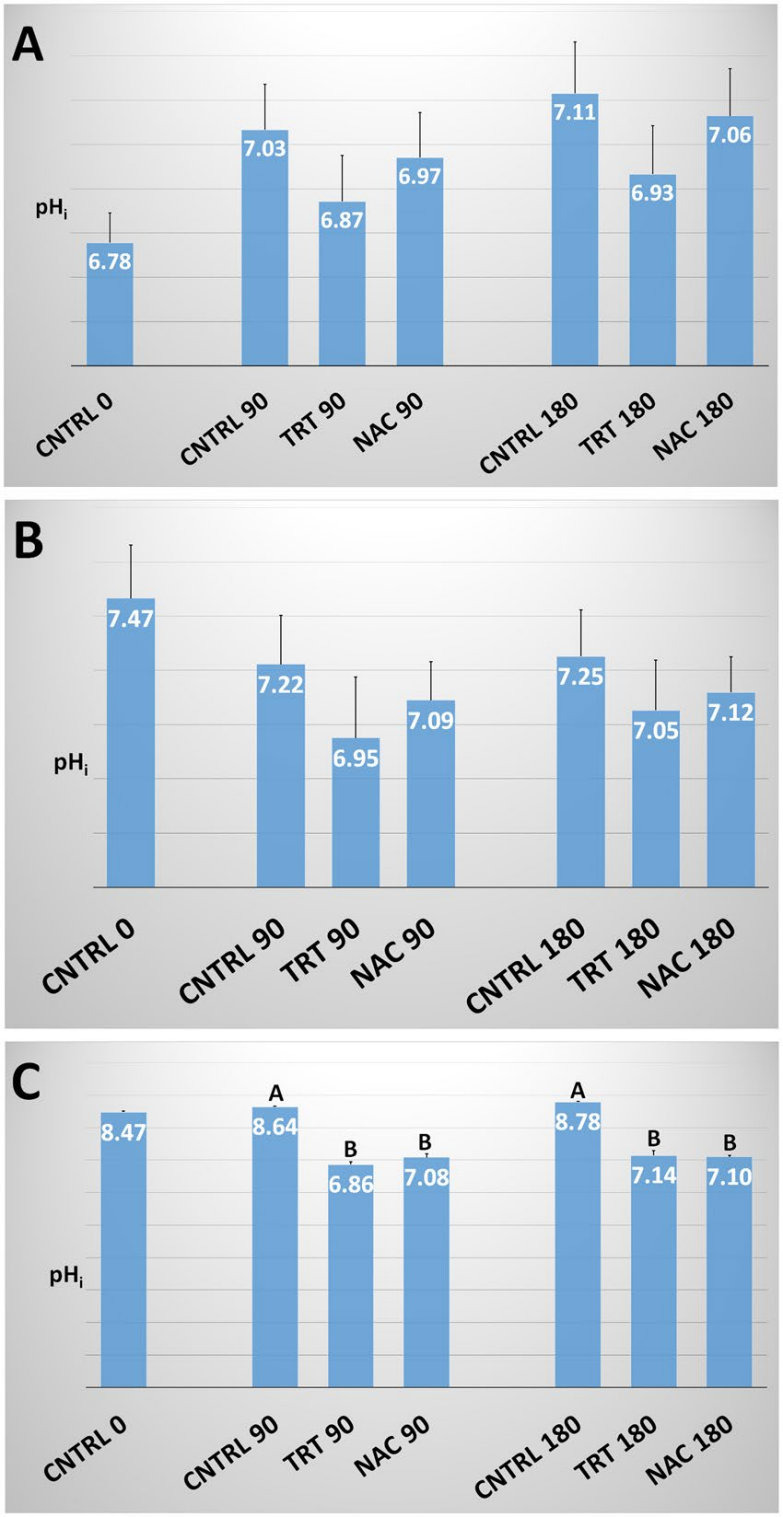
Intracellular pH in human, bovine and ascidian spermatozoa exposed to metabolic enhancers. Intracellular pH (pH_i_) in human (**A**), bovine **(B**) and ascidian (**C**) sperm divided in three groups treated with: (1) five metabolic enhancers (vitamins B6 and B12, 5 methyl THF, zinc and N-acetyl cysteine) (TRT group); (2) n-acetyl-cysteine (NAC group); and (3) medium alone (CNTRL group). Mean (±SE) values of pH_i_ in spermatozoa loaded with BCECF, arranged in TRT, NAC and CNTRL groups and then incubated for 0, 90 and 180 min. The fluorescence emission peaks (Fo) were measured at 535 nm after excitation at 440 and 490 nm. Statistically significant differences between groups for each evaluated time point are expressed as different capital (A *vs*. B; P < 0.01) letters.

**Figure 7:**
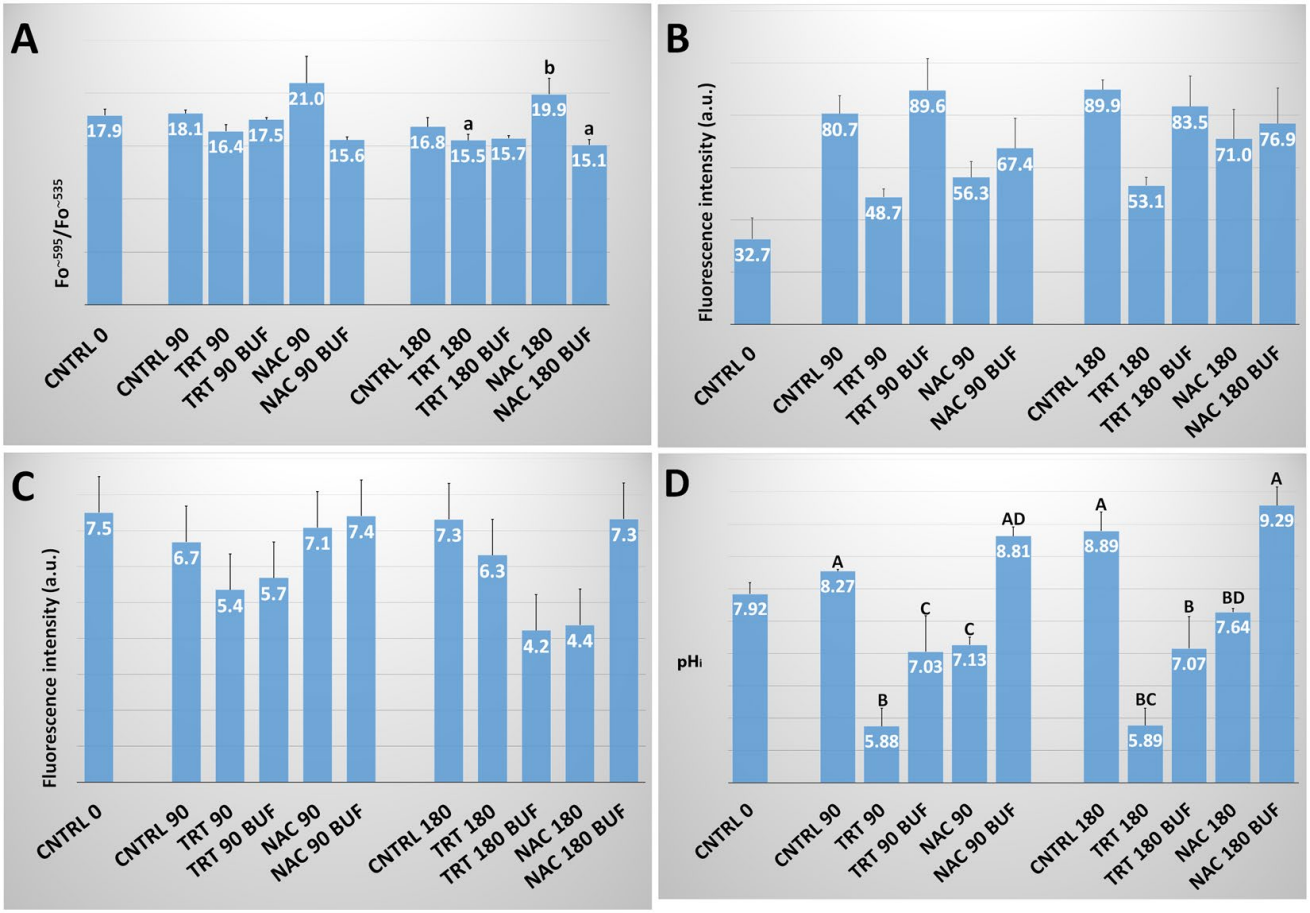
Effect of extracellular pH in ascidian spermatozoa exposed to metabolic enhancers. Mitochondrial membrane potential (ΔΨM) (**A**), Reactive Oxygen Species (ROS) detected by either H_2_DCFDA (**B**) or dihydroethidium (**C**) and intracellular pH (**D**) in ascidian sperm divided in five groups treated with: (a) five metabolic enhancers (vitamins B6 and B12, 5 methyl THF, zinc and N-acetyl cysteine) simply supplemented to FNSW (TRT group) and NFSW that was, after this supplementation, buffered at pH = 8.2 (TRT buf group); (b) n-acetyl-cysteine simply supplemented to FNSW (NAC group) and NFSW that was, after this supplementation, buffered at pH = 8.2 (NAC buf group); and (c) FNSW alone (CNTRL group). Mean (±SE) values obtained for each analytical determination were reported at 0, 90 and 180 min incubation. (n = 6 replications). Statistically significant differences between groups for each evaluated time point are expressed as different capital (A *vs*. B and C *vs*. D; P < 0.01) and small (a *vs*. b; P < 0.05) letters.

The pH_i_ in the CNTRL group in human and ascidian sperm significantly (P < 0.01) increased as a function of the time of incubation, whereas in the bovine sperm it maintained a stable pattern (Fig. 6). In human and bovine species, both treatments did not significantly affect pH_i_ although a small decrease of pH_i_ was found in both TRT and NAC groups with respect to the CNTRL group at either 90 or 180 min incubation. In ascidian sperm, a dramatic decrease of pH_i_ was observed in TRT and NAC groups at either 90 or 180 min incubation. Following the pH measurement of culture media used to incubate each species, we found a decrease of the pH value of 0.2–0.4 units when human and bovine culture media (i.e., SP and TRIS-fructose, respectively) were supplemented with either the five treatment compounds or the only NAC; however, this decrease was equivalent to 2.0–2.5 pH units in filtered seawater used to incubate ascidian spermatozoa. When FNSW was buffered to pH = 8.2 with NaHCO_3_ in the ascidian TRT or NAC groups, different results were obtained in relation to ΔΨM, pH_i_ and ROS production (Fig. 7). In particular, the ΔΨM increase found in NAC at 180 min incubation was annulled (P < 0.05) when FNSW was buffered (Fig. 7A); the production of ROS, detected by either H_2_DCFDA or DHE (Fig. 7B,C), did not show significant differences; the pH_i_ significantly increased in NAC group following FNSW buffering (Fig. 7D), however, in TRT-buffered group this increase did not significantly reset pH_i_ to CNTRL values at neither 90 nor 180 min incubation.

## Discussion

The progressive decline in human male fertility over the last years^22^ highlights the need for strategies to prevent further drops in sperm quality, which is especially manifest when treating males for assisted reproductive technologies. The 1-CC is an important target for maintaining sperm quality as it is involved in methylation and generation of three major antioxidants: hypotaurine, glutathione and Coenzyme Q10^23,24^. The 1-CC, which links folate and methionine cycles, underlies this effect in conjunction with the trans-sulfuration and BHMT pathways. The 1-CC and the associated folic acid pathway allow the regeneration of homocysteine to methionine and the synthesis of S adenosyl methionine (SAM). SAM is the universal methyl donor, the precursor of polyamines and a compulsory stabilizer of DNA^24^. The trans-sulfuration pathway, via the genesis of cysteine, enhances the synthesis of glutathione. In the present study, we tested the supplementation of sperm culture medium with some of the major effectors of 1-CC, i.e., Vitamins B6 and methyl B12, 5MTHF, NAC and Zinc. Furthermore, we focused on NAC that plays a special role in the production of glutathione and hypotaurine^25^.

These effects were evaluated together with standard computer-assisted sperm kinetics, by using a simple, accurate, rapid, and sensitive methodology, which combines fluorescent probes with spectrofluorimetric analysis for the assessment of ΔΨM, lipid peroxidation, ROS production and pH_i_ in mammalian (human and bovine), as well as in the marine invertebrate (*C. robusta*) spermatozoa. In human sperm, this methodology is a novelty whereas in bovine and ascidian sperm this methodological approach have been partially tested in recent studies^26–28^. Beneficial effects of NAC on semen parameters including sperm motility have been already described in several mammalian species, such as bovine^29^, human^30^ as well as in the rooster^31^.

The analysis of ΔΨM revealed that both the full mixture of enhancers or NAC alone have beneficial effects up to 180 min incubation in the two mammalian species. In ascidian sperm, only NAC showed a significant improvement of ΔΨM either at 90 min or 180 min incubation showing a clear discrepancy with respect to the combined treatment.

N-Acetylcysteine (NAC) improves sperm function^32^ and when combined with Acetyl-L-Carnitine and α-Lipoic Acid has a significant beneficial effect on *in vitro* fertilized mouse pronucleate oocyte development, especially under oxidative stress^33^. In addition, as demonstrated in H9c2 cells, it has an antiapoptotic activity against glucose/glucose oxidase-induced oxidative stress by the inhibition of mitochondrial damage and the maintenance of ΔΨM values^34^. The ΔΨM is generated by the electron-transport chain and regulated by an oxidation–reduction equilibrium of ROS, pyridine nucleotides (NADH/NAD^+^ and NADPH/NADP), and reduced glutathione (GSH)^35^. ΔΨM, together with the production of ROS, is crucial for the forward motility and fertility of spermatozoa^36–38^. Hence, the beneficial effects of NAC on ΔΨM was predictable as well as the combined treatment containing NAC. There was no additive effect in human or bovine sperm from the other four components, as demonstrated in preliminary trials of single compound exposure (Supplemental Fig. S2), where, unlike NAC, no significant effects were observed on ΔΨM values by each single supplementation. In ascidians, the lower ΔΨM values observed in the TRT group may be due to the negative effects of the four other components. The role of vitamins in marine organisms is, in fact, critical. In the marine systems, taxonomic changes observed in marine phytoplankton communities could be the result of their specific vitamin requirements and/or vitamin availability. Dissolved vitamin concentrations show that large areas of the world oceans are devoid of B vitamins, suggesting that vitamin limitation could be important for the efficiency of carbon and nitrogen fixation in these regions^39^. Consequently, the effects of adding vitamins to marine animals may depend on conditions particular to these marine organisms in nature.

Sperm lipid peroxidation is an effective indicator of sperm quality, oxidative stress and storage resistance^40^. Moderate ROS levels exert positive effects during sperm capacitation and fertilization^41^; however, high ROS levels cause sperm damage, affect all biomolecules and, ultimately, lead to a cellular stress or even death^42^. The lipophilic fluorescent probe C11-bodipy shows oxidative damage in cells by changing its fluorescence when it interacts with peroxyradicals^27^. In our study, we did not find differences between TRT, NAC and CNTRL groups in relation to peroxidation, which may be due to the short incubation time used; however, the progressive increase in lipid peroxidation with time of incubation suggests the experimentation was correct. This was further demonstrated by the use of positive controls, by adding 750 µM ascorbic acid and 150 µM FeSO_4_ to the sperm extender^27^.

ROS production was determined by evaluating either dichlorofluorescein (H_2_DCFDA) or 2-OH ethidium fluorescence by spectrofluorometric analysis. Dichlorofluorescein (H_2_DCFH) enters cells in the diacetate form (H_2_DCFH-DA). It is hydrolyzed and trapped as DCFH, a nonfluorescent compound. Subsequent oxidation of DCFH by H_2_O_2_, catalyzed by peroxidases, yields the highly fluorescent DCF^43^. This chemical reporter is widely applied as a “hydrogen peroxide (H_2_O_2_)-specific probe” in intact cells^44^. However, the range of ROS detected by this probe is much broader since DCF formation is also induced by other radical species including nitrogen derived free radicals^45,46^ rather than O_2_^−^^47^. However, the superoxide radical can be enzymatically dismutated to form H_2_O_2_ and further stimulate amplification of the DCF signal^48^. In our study, this ROS indicator shows clear species-specific modifications. In all the studied species, it increased along with incubation time. In human sperm, ROS levels appear higher in the NAC than in the CNTRL with an intermediate value in the TRT group. The high concentration of NAC (2 mM) may have exerted a paradox pro-oxidant effect via reduction of transition metal ions and enhancement of Fenton chemistry, as shown for high dose vitamin C^49^. This paradox effect was likely partially compensated by the antioxidant effect of other substances in TRT group. This was not seen in bovine sperm where the CNTRL group exhibits higher levels of ROS than treated groups at both 90 and 180 min of incubation. Indeed, bovine sperm unlike the other species have been subjected to freezing/thawing procedure, a particularly stressful condition that has led them to benefit from the antioxidant treatment^50^. In ascidian sperm, the situation is also different without significant differences between groups.

Dihydroethidium (DHE) is an intracellular indicator of the ROS superoxide ion quota^51^. This probe is able to detect a non-mitochondrial ROS source that cannot be disrupted by mitochondrial inhibitors such as rotenone and CCCP^52^. However, this hypothesis was revised when a derivative of DHE, MitoSOX Red, was developed. This probe contains a positively charged triphosphonium cation that allows it to concentrate in the mitochondrial matrix and efficiently detects mitochondrial-derived O_2_^−^ in human sperm^53^. More recently, Aitken *et al*.^54^ demonstrated in human sperm exposed to oxidative stress that DHE was highly correlated with MitoSOX Red (r = 0.993) for the evaluation of O_2_^−^ generation. In our study, the specificity of superoxide detection is confirmed by the analysis at spectrofluorometer of 2OH-Etidium in positive controls obtained by treating sperm samples for 1 hour with 30 µM pyrogallol, a potent superoxide ion generator^55^ (Supplemental Fig. S3). In all the studied species, we found a similar effect from the evaluated treatment. In particular, the combination of the five vitamin-antioxidant components linked to the 1-CC cycle significantly reduced the production of superoxide ions whereas NAC alone did not. This finding agrees with studies carried out in other species. In the horse, incubation of spermatozoa under both hyposmotic and hyperosmotic conditions increases superoxide anion generation; this production can be inhibited by tiron, a superoxide scavenger as well as MAP kinase p38 inhibitor, SB203580^56^.

No significant effects were observed on the intracellular pH_i_ of human and bovine sperm in relation to the treatments used. The high buffering capacity of the extenders used in human and bovine species may have counteracted the NAC-induced acidification found in ascidian sperm; this was based on the reduced buffering potential of natural sea water and was indeed reverted by buffering with NaHCO_3_. However, the NAC-induced acidification of the medium resulted to be the determining factor for the positive effects of treatment in this species. Actually, the fact that the NAC exhibits an isoelectric point equal to 5, makes the treatment more effective in acidic environments, canceling its effects under the typical alkaline conditions of ascidian sperm. In the human, previous studies^57^ found that 0.5 mM NAC prevented sperm immobilization induced by Stat3 inhibitory compound V (Stattic V) as well as the production of superoxide anion, mitochondrial membrane depolarization and the oxidation of protein free thiols caused by Stattic V; however, with regards to pH_i_, 0.5 mM NAC did not significantly affect pH_i_ but efficiently counteracted Stattic V-induced acidification.

Overall, compared to NAC only, the association of the other 1-CC enhancers (TRT) produced some further advantages. TRT groups showed lower production of ROS detected by DHE compared to CNTRL and NAC. TRT also partially restored the excess of ROS production measured by H_2_DCFDA in NAC group, a paradox pro-oxidant effect due to its high concentration. Indeed, the activation of the 1-CC serves as a homeostatic signal for the up-regulation of GSH synthesis^58,59^. This added antioxidant benefit may account for the TRT improvements over NAC alone on ROS production and may be very relevant in *in vivo* conditions. Indeed, NAC oral administration in humans generates circulating concentrations in the range of 5 µM, which is far below the concentration tested in this study and that are supposed to benefit only by inducing GSH synthesis without any real direct antioxidant effect^60^. Therefore, the positive *in vivo* effect of NAC is linked to the induction of GSH synthesis and it is predicted to benefit from an extended 1-CC support.

Although both the extenders used for human (SP) and bovine (Tris-fructose) semen contain components such as human serum albumin and insulin (SP) and bovine serum albumin (Tris-fructose) which could perform a *per se* antioxidant role and interact with the evaluated treatments, the use of these media is mandatory to assure optimal *in vitro* culture conditions of spermatozoa.

In conclusion, the proposed multiple vitamin/antioxidant treatment efficiently enhanced human and bovine sperm metabolic activity based on sperm kinetics and ΔΨM indications. Since there were no significant differences between the TRT and the NAC treatment alone, we may attribute these beneficial effects, except for the inhibition of ROS production, to NAC. The association of other 1-Carbon Cycle enhancers further improved the effect on sperm kinetics and ROS production indicating that they may be relevant in the clinical setting when the attainable concentration of NAC is a lot lower. The fast metabolism associated with the reduced cytoplasmic component typical of spermatozoon makes this specialized cell an unexpected user of the 1-Carbon Cycle. Ascidian *C. robusta* sperm showed lower advantages following the vitamin/antioxidant treatment that were, however, strictly dependent on the extra- and intra-cellular acidification induced.

## Materials and Methods

### Chemicals

Nigericin, Triton X-100, sodium citrate, HEPES, Penicillin-Streptomycin, pyridoxine (Vitamin B6), zinc bisglycinate, n-acetyl-cysteine, water and all the culture medium compounds were purchased by Sigma Chemical Company (Milan, Italy) and cell culture tested.

(6S)-5-methyltetrahydrofolate/glucosamine salt (5MTHF) and methylcobalamin (reduced vitamin B12) were kindly provided by Parthenogen, Switzerland. Carbonyl cyanide 3-chlorophenylhydrazone (CCCP), 5,5′,6,6′-tetrachloro-1,1′,3,3′-tetraethylbenzimidazolyl-carbocyanine iodide (JC-1), 4,4-difluoro-5-(4-phenyl-1,3,butadienyl)4-bora-3a,4a-diaza-s-indacene-33-undecanoic acid (C11-BODIPY^581/591^), 2′,7′-bis-(2-carboxyethyl)-5-(and-6)-carboxy-fluorescein acetoxymethyl ester (BCECF-AM), 2′,7′-dichlorodihydrofluorescein diacetate (H_2_DCFDA) and dihydroethidium (DHE) were obtained from Life Technologies (Milan, Italy).

### Human sperm preparation

Sperm samples were provided by patients, with written consent, attending to the Centre for Assisted Fertilization (CFA), Naples, Italy, between October 2016 and April 2017. The sperm used for experimentation was excess material not used for clinical purposes and was normally discarded. The experimentation on human spermatozoa was approved by the ethical committee of CFA. Each semen sample was evaluated for sperm concentration and motility by using a Makler chamber (Sefi Medical Instruments, Haifa, Israel). Only ejaculates from normospermic individuals according to WHO criteria^61^ were used for experiments.

Spermatozoa were isolated by centrifugation over 45% and 80% Percoll gradients for 30 min at 200 g. They were washed, resuspended in Sperm Preparation (SP) medium (Origio, DK) and evaluated for their concentration and motility.

### Bovine sperm preparation

Frozen bovine semen from three ejaculates of three Holstein Friesian bulls of proven fertility were used in all experiments and obtained from Inseme (Modena, Italy). For each experiment, straws of each bull were thawed in air (10 sec), then, in a water bath at 37 °C for 30 sec and mixed in a pool. Spermatozoa were isolated by centrifugation over 45% and 80% percoll gradient for 30 min at 200 g. They were washed in tris-fructose medium - i.e., 247.7 mM Tris (hydroxymethyl) aminomethane, 57.8 mM citric acid, 69.4 mM fructose, supplemented with 3 mg bovine serum albumin/mL and 1x Penicillin-Streptomycin solution in 1 L of final solution (pH = 7.5, 291 mOsm)^62^ - by centrifugation at 200 g for 10 min. Spermatozoa were resuspended in tris-fructose medium and evaluated for their concentration and motility.

### Ascidian sperm preparation

Adults of *C. robusta* were collected from the Gulf of Naples (Italy) and maintained in aquaria with running seawater at 18 °C for at least two days until the experiments. This ascidian is not protected by any environmental agency in Italy and Europe.

After anesthetization of animals on ice, spermatozoa were collected from sperm ducts. Sperm concentration and motility were evaluated by using a Makler chamber and, then, diluted to the desired concentration in filtered (Millipore 0.22 mm; MilliQ, Medford, MA) natural seawater (FNSW) (38 g/L salinity, pH 8.2 ± 0.1).

### Experimental design

Human, bovine and ascidian spermatozoa were suspended in their specific sperm extenders, i.e., SP and tris-fructose media and NFSW, respectively, and loaded with fluorescent indicators for the assessment of ΔΨM, pH_i_, lipid peroxidation and ROS production. Specific extenders were supplemented with: (1) five sperm metabolic enhancers, i.e., 60 µM pyridoxine, 40 µM 5MTHF, 7 µM methylcobalamin, 130 µM zinc bisglycinate and 2 mM n-acetyl-cysteine (treated group, TRT); (2) 2 mM n-acetyl-cysteine (n-acetyl-cysteine-treated group, NAC); (3) medium alone (control group, CNTRL). Fluorochrome-loaded spermatozoa were distributed in these three groups and incubated for 0, 90 and 180 minutes at 37 °C (human and bovine) and 18 °C (ascidian). All the sperm metabolic enhancers were water soluble; hence, 100X stock solutions of each compound were previously prepared and stored at −80 °C.

### Sperm kinetics

The assessment of the human and bovine sperm kinetics was carried out with a SCA 5.0 system (Microptic, Barcelona, Spain), whereas ascidian sperm kinetics were analyzed by CASA application of the open source ImageJ software. The extended spermatozoa were loaded into a Makler cell chamber and recorded using video cameras (CCD Hitachi, Tokyo, Japan and AxioCam 506, Ziess, Oberkochen, Germany). Sperm motility patterns were analyzed at 25 or 40 video frames per sec with the SCA and CASA systems, respectively. Sperm concentration was adjusted with specific extenders to a final concentration of 20 × 10^6^ spermatozoa/mL. The kinematic values recorded included: the overall percentage of motile spermatozoa; the curvilinear velocity (VCL, µm/s); the straight-line velocity (VSL, µm/s); the average path velocity (VAP, µm/s)^63^.

### Evaluation of Mitochondrial Membrane Potential (ΔΨM)

Sperm aliquots were diluted in their specific extender in order to obtain a concentration of approximately 1 × 10^7^ spermatozoa/mL and incubated with 5 µM JC-1 at room temperature (RT) for 30 min^13^. After incubation, sperm suspensions were centrifuged at 200 g for 10 min (human and bovine) at RT or at 800 g for 10 min at 4 °C (ascidian). Later, they were resuspended in their specific extender, divided in three aliquots at the same sperm concentration and arranged accordingly to CNTRL, TRT and NAC groups, as described above. In addition, one tube with the addition of CCCP to a final concentration of 2 µM served as a negative control^27^.

The fluorescence spectra were recorded in duplicate: 400 µL of each sample was placed in a quartz microtube (10 × 4 mm) high precision cell (Hellma Analytics, Müllheim, Germany) and read with a spectrofluorometer (Shimadzu RF-5301, Tokyo, Japan). The excitation wavelength was set at 490 nm, and the emission spectrum was recorded in the range of 500–650 nm (Supplemental Fig. S3). The ΔΨM was measured by evaluating the ratio of the fluorescence values at ~595 nm and ~535 nm.

### Evaluation of Intracellular pH (pH_i_)

Sperm suspensions (1 × 10^7^ spermatozoa/mL) of each species were incubated with 5 μM BCECF-AM, for 30 min at 37 °C (human and bovine) or 18 °C (ascidian) and, then, washed by centrifugation, as referred above. Later, spermatozoa were incubated for an additional 30 min to allow BCECF de-esterification. The spermatozoa were then washed, suspended in their specific extender and divided at the same sperm concentration in three groups. Aliquots of each sperm suspension were sampled, mixed and equally divided into three tubes containing the calibration solution (143 mM KCl, 5 mM HEPES, 290 mOsm) at different pH values supplemented with nigericin^64,65^.

At the end of each incubation time and at time 0 in the control group, samples were read with a spectrofluorometer, as described above. Data were obtained by dividing the emission intensity at 535 nm after excitation at 490 nm by the emission intensity at 535 nm after excitation at 440 nm. A linear-regression analysis of the calibration produced a formula that was used to obtain pH_i_ values for each experiment.

### Evaluation of ROS production

ROS production was determined by spectrofluorometric analysis evaluating either H_2_DCFDA or DHE fluorescence spectra. H_2_DCF has very low reactivity toward superoxide radicals whereas hydrogen peroxide indirectly facilitate its oxidation^48^.

DHE is a cell-permeable compound that, in the presence of superoxide anion, is oxidized to unspecific products, as ethidium, and a single superoxide-specific product, 2-OH-ethidium (2-OH E)^51,66^. For the H_2_DCFDA examination, sperm suspensions (2 × 10^7^ spermatozoa/mL) were incubated for 30 min at 37 °C (human and bovine) or 18 °C (ascidian) with 10 µM H_2_DCFDA. Then, spermatozoa were washed and incubated as described above for additional 30 min. Later, spermatozoa were washed and divided in tubes containing the specific extenders arranged for the three experimental groups and the three times of evaluation. Positive control was obtained by incubating 1 h samples of sperm suspension with 25 µM H_2_O_2_. Samples were read with a spectrofluorometer, as described above. The excitation wavelength was set at 490 nm, and the emission spectrum was recorded at 500–560 nm (Supplemental Fig. S3). ROS production was evaluated as arbitrary units of fluorescent signal.

For the DHE protocol, sperm suspensions (30 × 10^7^ spermatozoa/mL) of the three experimental groups, at the end of each incubation time and at time 0 in the control group, were incubated for 30 min at 37 °C (human and bovine) or 18 °C (ascidian) with 20 µM DHE. Positive control was obtained by incubating samples of sperm suspension with 30 µM pyrogallol together with DHE.

At the end of the incubation time, samples were read with a spectrofluorometer, as described above. The excitation wavelength was set at 350 nm, and the emission spectrum was recorded in the range of 550–670 nm (Supplemental Fig. S3). ROS production was evaluated as arbitrary units of fluorescent signal.

### Evaluation of Lipid Peroxidation

Lipid peroxidation was evaluated by C11- BODIPY^581/591^ fluorescence and spectrofluorometric analysis^67^. Sperm aliquots (2 × 10^7^ spermatozoa/mL) were incubated with 5 µM C11-BODIPY^581/591^ for 30 min at 37 °C (human and bovine) and 18 °C (ascidian). The spermatozoa were then centrifuged and resuspended in each specific extender. Aliquots of each sample were mixed and used as a positive control which involved incubating for 1 h with 150 µM FeSO_4_ and 750 µM ascorbic acid before spectrofluorometric evaluation^27^.

After incubation, aliquots of each sample were read with a spectrofluorometer, as described above. The excitation wavelength was set at 490 nm, and the emission spectrum was recorded in the range of 500–650 nm. Lipid peroxidation was evaluated by relating the fluorescence peak values at ~520 nm to the sum of the fluorescence peak values at ~520 and ~595 nm (Supplemental Fig. S3).

### Confocal laser scanning microscopy analysis

Each fluorochrome used in this study was first evaluated by confocal laser scanning microscopy (Zeiss LSM 510) to assess its localization within a specific cell compartment (Supplemental Fig. S4).

### Statistical analysis

Data were obtained in six to eight replicates and entered in a spreadsheet as follows: the replication number, the time of incubation, the three experimental groups (TRT, NAC and CNTRL), and the sperm parameters evaluated, i.e., total sperm motility and kinetics, ΔΨM, pH_i_, ROS production by either H_2_DCFDA or DHE evaluations and lipid peroxidation. Before the analyses, percentage values were transformed in arcsin whereas, for pH_i_, H^+^ concentrations were log transformed. Repeated measures ANOVA (Systat 11.0) was used for evaluating either the time of incubation or treatment effects for each sperm parameter evaluated. Shapiro-Wilks test was used to evaluate the normal distribution of the data. Levene test was used to verify homogeneity of variances. Log transformation was applied when the normality hypothesis was rejected. Pair-wise comparisons of the means were performed with Bonferroni test.

## Electronic supplementary material

Supplementary Information
