# Supplementary material for: Metabolic enhancers supporting 1-carbon cycle affect sperm functionality: an in vitro comparative study
Source: Sci Rep. 2018 Aug 6;8:11769. doi: 10.1038/s41598-018-30066-9 (PMC6079007; doi:10.1038/s41598-018-30066-9)
Supplement: Supplementary file 1 — Supplementary Information [file 41598_2018_30066_MOESM1_ESM.docx]

**Supplementary Information for**

**Metabolic enhancers supporting 1-carbon cycle affect sperm functionality: an in vitro comparative study**

Alessandra Gallo, Yves Menezo, Brian Dale, Gianfranco Coppola, Maurizio Dattilo, Elisabetta Tosti, Raffaele Boni

**Correspondence to:** [raffaele.boni@unibas.it](mailto:raffaele.boni@unibas.it)

**This PDF file includes:**

Figs. S1 to S4

**Figure S1.** **Schematic map drafting the connection between the one-carbon cycle (1-CC) and the trans-sulfuration pathway (glutathione synthesis).** The methyl group of methionine is activated by adenylation to form S-Adenosyl-Methionine (SAM) that acts as the universal methyl donor for any acceptor including DNA. Following demethylation, SAM becomes S-adenosylhomocysteine (SAH) and then homocysteine. Homocysteine can be either remethylated from folates or betaine or enter the trans-sulfuration pathway for the synthesis of glutathione.


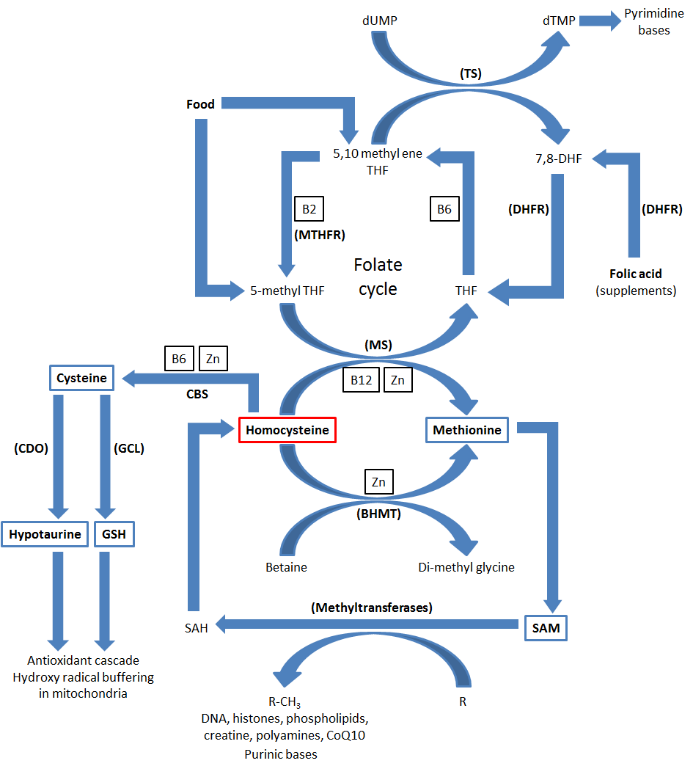


**Legend:** dUMP = deoxyuridin monophosphate; dTMP = deoxypyridine monophosphate: DHF = Dihydrofolate; THF = Tetrahydrofolate; SAH = S-Adenosyl-Homocysteine. Blue boxes represent metabolic effectors: SAM = S-Adenosyl-Methionine; GSH = Glutathione. Black boxes represent the essential co-factors to the main enzymes: B2, B6 and B12 = Vitamins B2, B6 and B12; Zn = Zinc. Acronyms in brackets/bold represent the main enzymes: TS = Thymidilate Synthase; DHFR = Di-Hydro-Folate-Reductase; MTHFR = Methyl-Tetra-Hydro-Folate-Reductase; MS = Methionine Synthase; BHMT = Betaine-Homocysteine-Methyl-Transferase; CBS = Cystathionine-Beta-Synthase; CDO = Cysteine-Di-Oxygenase; GCL = Glutamate-Cysteine-Ligase.

**Figure S2. ΔΨM and** **Lipid Peroxidation in bovine spermatozoa exposed to metabolic enhancers.** Mitochondrial membrane potential (ΔΨM) and lipid peroxidation in bovine spermatozoa at time 0 (CNTRL 0) and after 90 min. incubation with: (i) medium alone (CNTRL 90); (ii) 40 µM 5 methyl THF (5 methyl THF 90); (iii) 7 µM methylcobalamin (B12 90); (iv) 60 µM pyridoxine (B6 90); (v) 130 µM Zinc bisglycinate (Zinc 90); (vi) 2 mM n-acetyl-cysteine (NAC 90); (vii) all the above metabolic enhancers (vitamins B6 and B12, 5 methyl THF, zinc bisglycinate and n-acetyl-cysteine) at the dosages reported above (TRT 90); (viii) all the above metabolic enhancers without NAC (vitamins B6 and B12, 5 methyl THF and zinc bisglycinate) at the dosages reported above (TRT-NAC 90). Mean (±SE) values of ΔΨM in spermatozoa loaded with JC-1 (**A**) whose fluorescence emission peak (Fo) was measured at ~595 and ~535 nm and expressed as Fo^~595^/Fo^~535^. Lipid peroxidation was calculated in spermatozoa loaded with C11-BODIPY^581-591^ (**B**) by relating the emission peak (Fo) at ~520 nm to the sum of the fluorescence emission peaks at ~520 and ~595 nm, i.e., ((Fo^~520^/(Fo^~520^+ Fo^~595^))*100. Statistically significant differences between groups at 90 min incubation are expressed as different letters (A vs. B; P< 0.01). Number of replicates=3

| **A**  **B** | **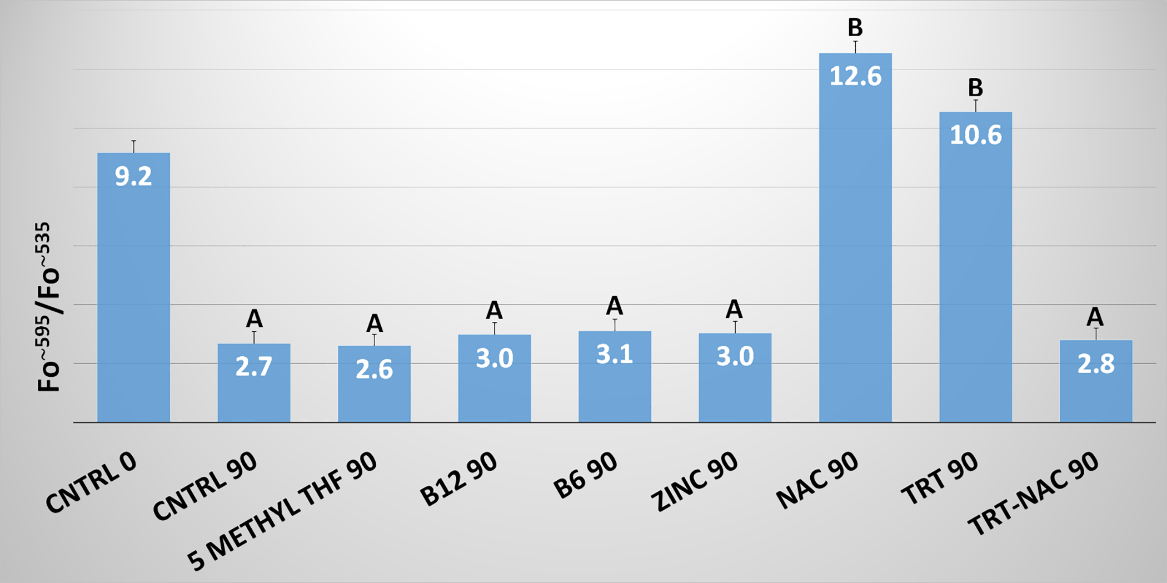**  **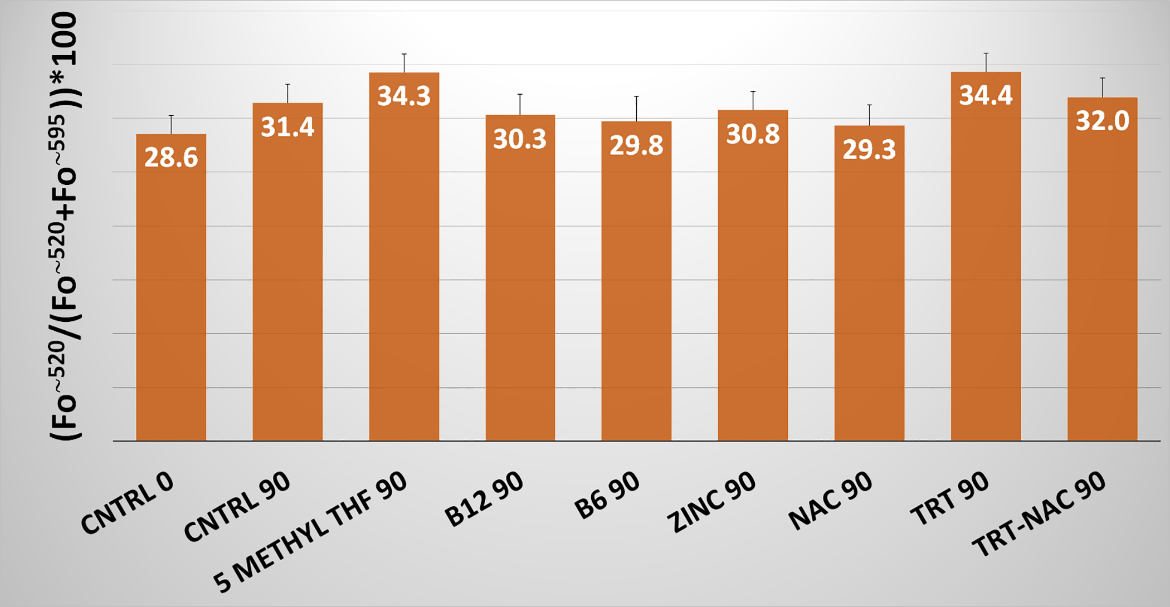** |
| --- | --- |

**Figure S3. Emission spectra of bovine sperm loaded with specific fluorescent dyes and their respective positive or negative controls.** Typical emission spectral diagrams recorded in frozen/thawed bovine sperm loaded with different fluorochromes, i.e., JC1 (**A**), C11- BODIPY^581-591^ (**B**), H_2_DCFDA (**C**) and DHE (**D**), and incubated 1 hour with specific compounds used as negative or positive controls, i.e., 2 µM CCCP (**A**), 150 µM FeSO4 and 750 µM ascorbic acid (Vit C)(**B**), 25 µM H_2_O_2_ (**C**), and 30 µM pyrogallol (**D**).

**
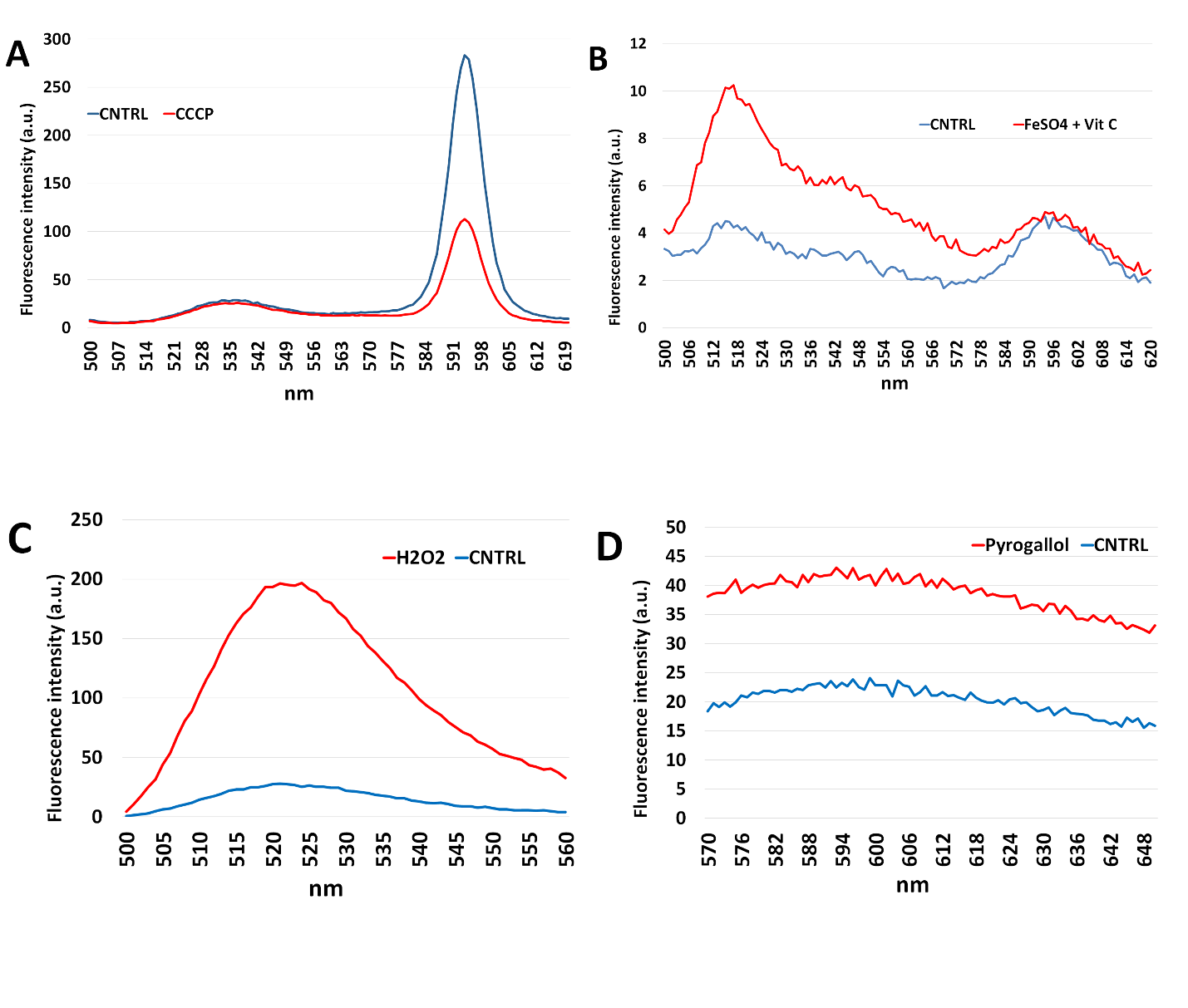
**

**Figure S4. Bovine spermatozoa loaded with ΔΨM, lipid peroxidation, ROS and pH_i_ indicators.** Representative images of bovine spermatozoa analyzed by laser confocal microscope for mitochondrial membrane potential (**A**), lipid peroxidation (**B**), H_2_DCFDA-detected (**C**) and 2-OH ethidium-detected (**D**) ROS production and intracellular pH (**E**). Nuclei were stained blue by 1 µg/mL DAPI, except than in panel D.

**
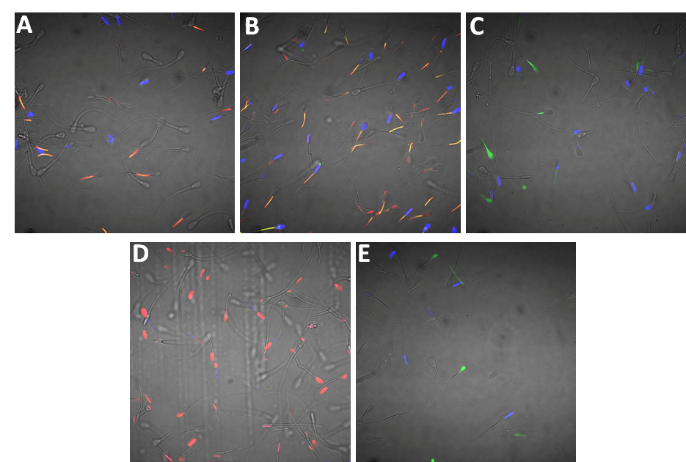
**
